# Surface Modification of Polymethylmethacrylate (PMMA) by Ultraviolet (UV) Irradiation and IPA Rinsing

**DOI:** 10.3390/mi13111952

**Published:** 2022-11-11

**Authors:** Geundong Bae, Taehyun Park, In-Hyouk Song

**Affiliations:** 1 School of Mechanical Engineering, Kyungnam University, Changwon 51767, Korea; 2 Department of Engineering Technology, Texas State University, San Marcos, TX 78666, USA

**Keywords:** surface modification, PMMA, IPA rinsing, UV irradiation, contact angle

## Abstract

Polymethylmethacrylate (PMMA) is commonly applied to microfluidic devices due to its excellent biocompatibility, high optical transparency, and suitability for mass production. Recently, various surface treatment methods have been reported to improve the wettability of polymers, which is directly related to adhesion. In this research, the effect of a UV irradiation technique and an IPA rinsing technique as surface treatments for PMMA is investigated regarding the water contact angle of the PMMA surface. PMMA sheets that were 1.62 mm thick and commercially available were exposed to UV light with four different exposure times. Significant decreases in the water contact angle were observed after exposure to UV light, and the lowered contact angles due to the UV irradiation increased over time. According to the measurement, the water contact angle is a function of UV exposure dose as well as storage time after UV exposure. We examined the effect of a IPA rinsing process after UV irradiation and observed an increase in the water contact angle.

## 1. Introduction

Polymers are the most common materials in industries with medical, construction, energy, water treatment, and electronic applications [1,2]. In particular, thermoplastic polymers such as polystyrene (PS), polymethyl methacrylate (PMMA), and cyclic olefin copolymer (COC) have become a material mainly used for microfluidic devices due to advantages such as transparency and biocompatibility [3,4,5,6,7,8]. Research to obtain surface properties suitable for each application has been actively carried out while maintaining the advantages of polymer [9,10,11]. In addition, surface modification techniques have broadened the applications and have been used effectively [12,13,14,15,16,17,18].

In this study, Polymethylmethacrylate (PMMA) was the focus, which is one of the most common plastics for microfluidic devices due to its excellent biocompatibility, high optical transparency, and suitability for mass production [19,20,21]. The wettability of polymers is an important property directly related to adhesion, colorability, biocompatibility, and electrical properties. Various surface treatment methods, such as physical, chemical, plasma, corona, annealing, and patterning, have been developed and reported to increase the surface energy of PMMA to enhance the wettability of PMMA and to positively affect its adhesive characteristics [12,22,23,24,25,26,27].

Surface treatments modify the surface energy of PMMA to be lower or higher depending on the specific applications. Photochemical oxidation is a technique using ultraviolet (UV) irradiation to remove organic contaminates from polymer surfaces and to improve adhesion. UV irradiation causes the surface of PMMA to transform into the carbonyl and carboxyl hydrophilic groups by reacting with oxygen or ozone [28,29]. In the case of PMMA irradiated in UV treatment, the hydrophobic surface becomes more hydrophilic. It has been reported that this hydrophilicity of the PMMA surface depends on irradiation time and storage duration after UV exposure [16,30,31,32]. However, limited studies have been devoted to understanding the surface properties of PMMA with UV exposure only. This paper aims to address the change in the wettability of PMMA surface as a function of the UV exposure dose, the long-term effect of PMMA surface after UV exposure and the restoration of surface property by rinsing with IPA after UV exposure. In this work, we studied the stability of UV-treated PMMA under different storage times up to 30 days.

## 2. Ultraviolet (UV) Surface Treatment

Ultraviolet (UV) surface treatment is an effective, inexpensive, and efficient non-contact polymer modification method with a few process steps only for functionalizing the surface [6,33]. In general, the extent to which UV light penetrates the surface of PMMA and modifies the surface properties is determined by exposure conditions such as wavelength, intensity, and exposure duration. In the case of polymer, the surface energy is low and the adhesion quality is poor due to weak attraction forces. UV irradiation, however, increases surface energy, making the surface hydrophilic. The hydrophilic surface enhances cell adhesion and cell growth on polymer surface, allowing the polymer to be applied to microfluidic devices in biomedical applications.

**Figure 1:**
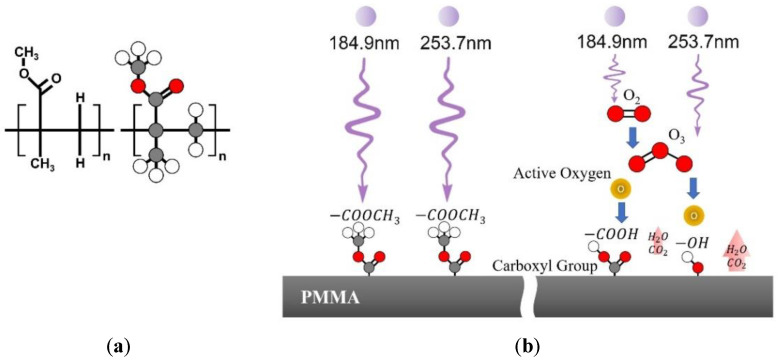
(**a**) Chemical structure of Polymethylmethacrylate (PMMA), (**b**) mechanism of PMMA surface modification by UV treatment.

As seen in Figure 1a, PMMA consists of carbon–carbon single bond and carbon–oxygen bond, carbonyl group and carbon-hydrogen bond (C-C, C-O, C=O, and C-H). The bonding dissociation energies of the chemical bonds of C-C, C-O, C=O, and C-H are 347.7 kJ/mol, 351.5 kJ/mol, 724 kJ/mol, and 413.4 kJ/mol, respectively [34]. That is, a dissociation energy of 724 kJ/mol is required to break C-C, C-O, C=O, and C-H bonds of the PMMA surface. UV is a form of electromagnetic radiation with a wavelength from 10 nm to 400 nm. The photon energy is given by $E=N\cdot h\cdot \frac{c}{\lambda }$, where *N* is Avogadro’s constant (6.022 × 10^23^ [mol^−1^]), constant *h* is Planck’s constant (6.626 × 10^−34^ [J·s]), *c* is the speed of light (2.998 × 10^8^ [m/s]), and *λ* is a wavelength of light [35]. The photon energies with wavelengths of 184.9 nm and 253.7 nm used for this experiment are 647 kJ/mol and 472 kJ/mol, respectively, which are higher than the bonding energy of single bonds such as C-C, C-O, and C-H. Hence, UV irradiation is able to break single bonds, such as C-C, C-O, and C-H of the surface of PMMA.

Figure 1b illustrates the mechanism of PMMA surface modification with UV exposure. UV irradiation at 184.9 nm reacts with atmospheric oxygen to form highly reactive atomic oxygen and ozone. The ozone then absorbs 253.7 nm photons and dissociates into O_2_ and atomic oxygen. The PMMA surface is oxidized by active oxygen, O_2_ and O_3_ through the hydrogen atom abstraction from the polymer chains. This causes the generation of a carboxyl group on the PMMA surface, increasing hydrophilicity and enhancing wettability. Atoms chemically broken by UV irradiation are volatilized into the atmosphere in the form of H_2_O or CO_2_ after bonding with active oxygen.

## 3. Experimental Setup

In this study, the changes in surface wettability of PMMA due to UV exposure time (or exposure dose), storage time, and rinsing effect with IPA after UV irradiation were studied. First, 1.62 mm thick PMMA sheets were used for this experiment. For cleaning, the sheets were immersed in a 10:1 diluted solution of DI water and IPA and washed in an ultrasonicator (Branson 5510R-DTH ultrasonic cleaner) to remove grease contaminants on the surface of PMMA for 3 min. After cleaning, moisture on the cleaned PMMA sheets was blown off using an air compressor. The sheets were then placed in a forced convection oven (JeioTech OF-02PW) and dehydrated at 60 °C for at least 5 h.

**Figure 2:**
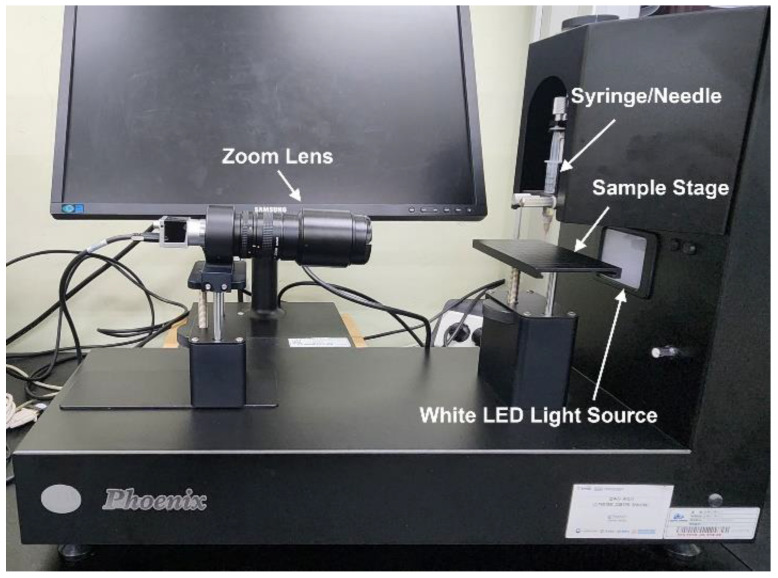
Contact angle analyzer, SEO—Phoenix 10 from S.E.O. Co. Ltd. (Seoul, Korea).

In order to study the change in the surface property of PMMA by UV irradiation, water contact angles of the cleaned PMMA sheets were measured with a contact angle analyzer (SEO—Phoenix 10, S.E.O. Co., Ltd., Suwon City, Korea), shown in Figure 2. The apparent contact angles of DI water solutions on PMMA sheets were measured via the sessile drop method. The PMMA sheet was placed on the sample stage where the contact angle would be measured without an inclination and allowing a clear image. The droplet volume used for each measurement was 16.5 µL. A static contact angle method was used to measure the contact angle. Twelve droplets were performed on each sheet. The reported results were the mean values from more than 50 measurements. All experiments were carried out at 20 °C. The trimmed mean values were used to obtain the average of the measured values of the water contact angles.

**Figure 3:**
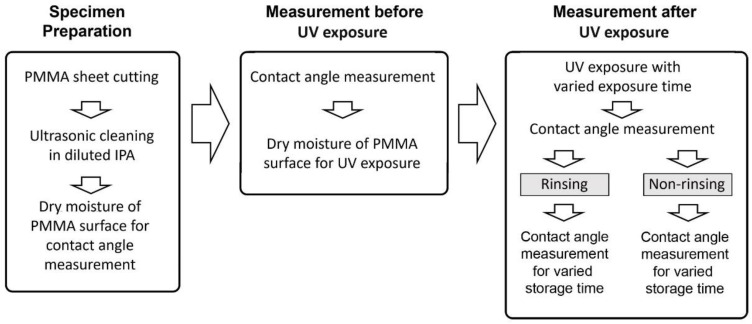
The Schematic Diagram of The Experimental Process.

To perform the surface treatment, the PMMA sheets were placed in a closed box and exposed to UV light. The intensity of UV irradiation at a wavelength of 253.7 nm was measured to be 5 mW/cm^2^. To study the relation between UV exposure dose and surface wettability, the samples were exposed for 5 min, 10 min, 20 min, and 30 min under a stabilized UV lamp, which are equivalent with the doses of 1.5 J/cm^2^, 3 J/cm^2^, 6 J/cm^2^, and 9 J/cm^2^, respectively. Figure 3 shows the schematic procedures of the experiment to collect data on the wettability of a PMMA surface for different surface treatment conditions.

## 4. Results and Discussion

The samples, classified into different exposure times—5 min, 10 min, 20 min, and 30 min—were stored in air for 1 h, 24 h, 7 days, 15 days, and 30 days, and the contact water angles were measured to investigate the long-term effect of surface properties. Additionally, the experiment was divided into two sets: (1) non-rinsing after UV exposure, (2) rinsing with IPA and DI water after UV exposure to examine the rinsing effect after UV irradiation. The IPA rinsing process was carried out immediately after UV irradiation, and the water contact angles of the rinsed samples were additionally measured.

**Figure 4:**
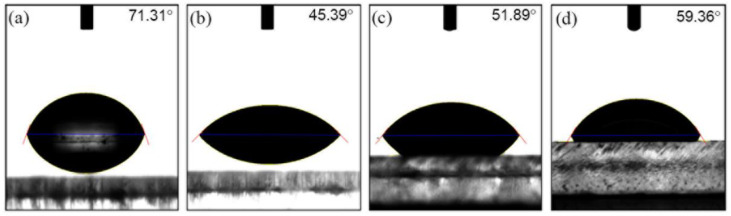
Water drops and contact angles on PMMA surface for different storage times: (**a**) for pristine: 71.31°, (**b**) right after UV exposure of 6 J/cm^2^: 45.39°, (**c**) 24 h after UV exposure of 6 J/cm^2^: 51.89°, (**d**) 30 days after UV exposure of 6 J/cm^2^: 59.36°.

The water contact angle here is the angle where the liquid interface meets PMMA surface. When the cohesive force of the liquid molecules is greater than the adhesive force between the liquid molecules and the PMMA surface molecules, the contact angle increases. That is, the wettability of the surface of the solid can be confirmed by the contact angle. Figure 4 shows the water drop images on the PMMA surface exposed to UV light for 6 J/cm^2^. Figure 4a shows the water contact angle of non-UV exposure, and Figure 4b shows the contact angle right after 20 min of UV irradiation. The contact angle was shifted from 71.31° to 45.39°, which means the surface of PMMA becomes more hydrophilic. However, the contact angles were increased as the storage time is longer. It was 51.89° after 24 h (Figure 4c) and 59.36° after 30 days (Figure 4d). Table 1 shows the mean values of the water contact angles from more than 50 measurements with standard deviation for non-rinsing samples with different doses as a function of storage durations. To eliminate the influence of outliers and to remove certain erratic observations, the trimmed mean values of collected data were used for this research.

**Figure 5:**
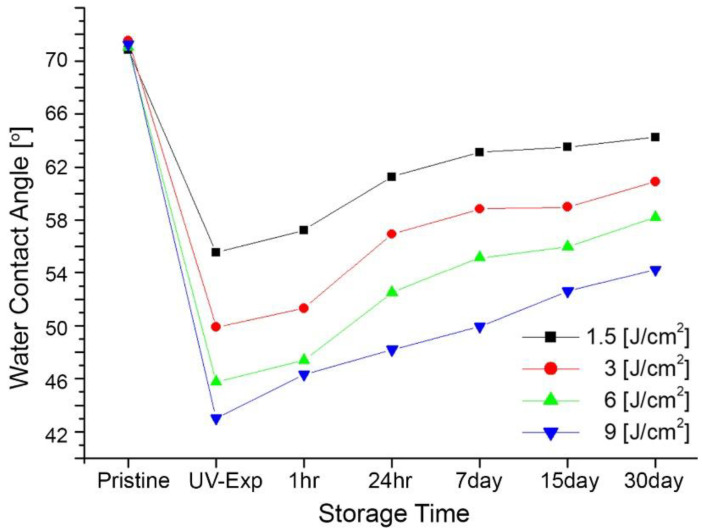
Average change in water contact angles of PMMA surface by storage time after UV exposure without IPA rinsing.

Figure 5 shows the changes in water contact angles as a function of UV exposure dose and storage times up to 30 days for non-rinsing samples after UV exposure. The water contact angles decreased when increasing UV exposure dose. For example, the angle was approximately 71° for pristine. The larger the UV exposure dose, the smaller the surface tension is. For example, the angles are 55.57°, 49.89°, 45.75°, and 43.03° for UV doses of 1.5 J/cm^2^, 3 J/cm^2^, 6 J/cm^2^, and 9 J/cm^2^, respectively, as shown in Figure 5. For all cases, the lowered surface tension of PMMA becomes larger over time. In other words, the smaller the wetting tendency, the longer the storage time is.

**Figure 6:**
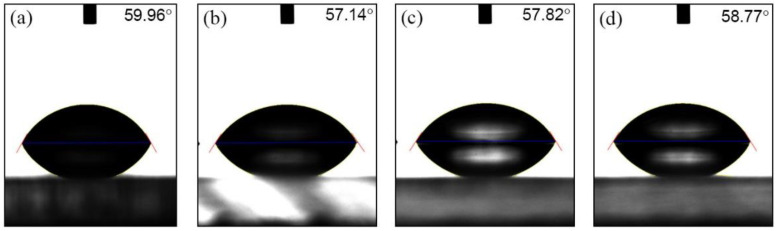
Water drops and contact angles on the surface of IPA-rinsed PMMA after UV irradiation: (**a**) UV dose of 1.5 J/cm^2^: 59.96°, (**b**) UV dose of 3 J/cm^2^: 57.14°, (**c**) UV dose of 6 J/cm^2^: 57.82°, (**d**) UV dose of 9 J/cm^2^: 58.77°.

**Figure 7:**
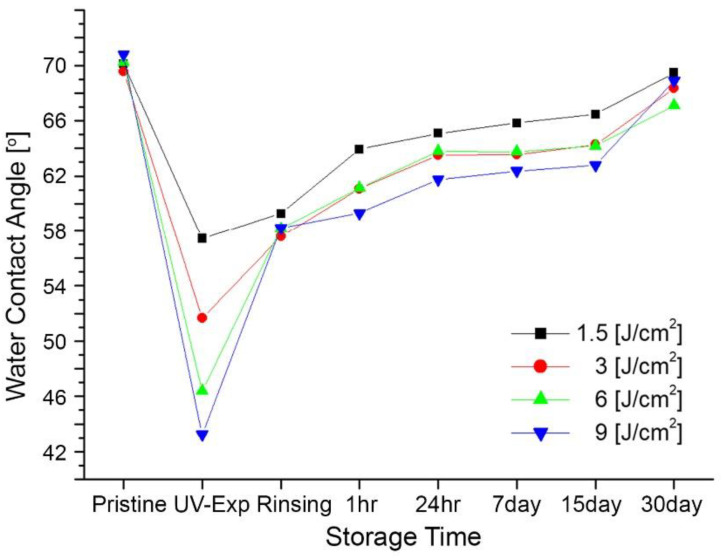
Average change in water contact angles of PMMA surface by storage time after UV-exposure for IPA-rinsed PMMA sheets.

In order to evaluate the surface wettability of a rinsing effect with IPA after UV exposure, the samples were rinsed with IPA and DI water after UV irradiation. The contact angles of the rinsed samples were measured with the same method we used for non-rinsed samples. Figure 6 shows the water contact angles of 59.96°, 57.14°, 57.82°, and 58.77° for UV doses of 1.5 J/cm^2^, 3 J/cm^2^, 6 J/cm^2^, and 9 J/cm^2^ right after rinsing with IPA and DI water, which were 55.74°, 52.21°, 46.46°, and 44.19°, respectively, before the IPA rinsing process. The contact angles increased after the PMMA sheets were rinsed with IPA and DI water. Table 2 shows the mean values of the water contact angles from more than 50 measurements, with the standard deviation for IPA rinsed samples with different doses and as a function of storage times. Regardless of the exposure dose, the contact angles of the rinsed samples were significantly increased compared to that of the non-rinsed sample. After UV irradiation, the surface tension can be lowered or increased with chemical treatment [16,18,33,36]. Scheicher reported that an IPA rinsing causes a significant loss in functional groups, especially carboxylic acid groups generated during UV irradiation [18]. The loss of these functional groups increases the water contact angle on the UV-treated surfaces, which is in good agreement with the previous literature data on IPA rinsing after UV irradiation [16]. For example, the average contact angle of 30 min exposure time samples, which was 43.2° ± 1.3° before rinsing, changed to 58.2° ± 2.4° after rinsing. Figure 7 shows the change in contact angles over time. Similarly to the non-rinsed PMMA sample, the contact angles of the IPA-rinsed samples increased over time.

As shown in Table 1 and Table 2, the water contact angles of UV-treated surface were restored over time. The change in the contact angles of non-rinsed PMMA samples and IPA-rinsed PMMA samples were observed in increments of 1.1~4.7°/h after surface treatment. The increment rates of the contact angle were of 3.5~7.0°/day during 24 h after surface treatment, and the contact angle changes were slowed to 0.02~0.4°/day after 24 h by 30th day measurements. In other words, the surface recovery rate is fast during the first hour and slows down over time.

**Figure 8:**
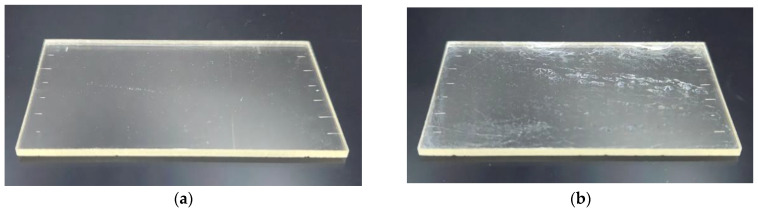

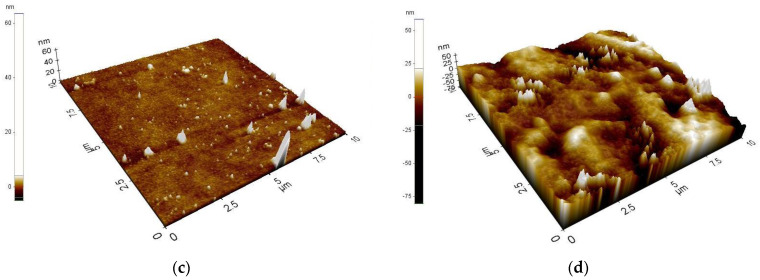
(**a**) Non-rinsed PMMA sheet after 30 min of UV exposure. (**b**) Rinsed PMMA sheet with IPA after 30 min of UV exposure. (**c**) AFM 3D scan image of PMMA surface of (**a**). The measured rms roughness is 0.6 nm. (**d**) AFM 3D scan image of the surface of (**b**). The measured rms roughness is 8.62 nm.

In the case of a sample exposed to UV for 30 min, the surface of PMMA is excessively modified with carboxyl hydrophilic group (-COOH), and damage occurs during the IPA rinsing process. Most polymers are susceptible to damage by UV since they consist of covalently bonded organic constituents. The UV damage mechanism in polymers is chain scission by photolysis. That is, long chains are cleaved into shorter ones by the direct action of UV photons. The absorption of high-energy UV photons can dissociate chemical bonds, resulting in a degradation of physical properties such as strength and ductility. A physically weakened surface is easily damaged chemically by IPA solution. Figure 8a,b are the images of a pre-IPA rinsing sample and a post-IPA rinsed sample of UV exposure dose of 9 J/cm^2^, respectively. Figure 8b clearly shows the damaged surface of the PMMA sheet during IPA rinsing. The surfaces of both PMMA sheets were profiled using an atomic force microscopy (AFM) in contact mode, and surface roughness was evaluated. AFM measurements performed on a 10 µm × 10 µm area were averaged to provide a statistical estimation of the true surface roughness. Figure 8c,d show 3D AFM surface images of Figure 8a, the pre-IPA rinsing sample, and Figure 8d, the post-IPA rinsed sample, respectively. While the average rms roughness value of the pre-IPA rinsing sample was 0.61 ± 0.09 nm, the surface roughness was increased to 8.37 ± 0.25 nm after IPA rinsing process.

## 5. Conclusions

The surface treatment technique using UV irradiation is widely used in polymer bonding for microfluidic device applications since the process is simple and inexpensive, and it is possible to treat large areas simultaneously. The advantage of the UV surface treatment method is that it is clean and produces little waste, unlike the wet chemical surface treatment method. The surface treatment of PMMA by UV irradiation was characterized by measuring the water contact angles of PMMA surface. The experiment results showed that UV irradiation imparts hydrophilicity to the hydrophobic PMMA surface. An important process parameter was found to be UV exposure dose. The higher the UV exposure dose, the higher the wettability. That is, the surface energy was raised by increasing UV exposure dose (or UV exposure time). However, if the surface is required to expose the IPA solution after UV irradiation, the weakened boundary layer of PMMA due to a high UV exposure dose may be damaged and roughened during the IPA rinsing process. Hence, it is necessary to determine an appropriate exposure time considering the possibility of the surface damage in subsequent processes.

To investigate the long-term effects of the modified surface energy over storage times, the contact angles were measured right after UV exposure, 1 h, 24 h, 7 days, 15 days, and 30 days. The contact angles increased over time. The surface energy, raised through the surface treatment process, was restored, and the water contact angles increased by 8.6°, 11.0°, 12.4°, and 11.2° for UV doses of 1.5 J/cm^2^, 3 J/cm^2^, 6 J/cm^2^, and 9 J/cm^2^ from the initial values for 30 days. For microfluidic applications, a sealing process is required to enclose microfluidic channels. The sealing process is one of the crucial and challenging steps required to complete polymer-based microfluidic devices and needs to enclose the channel tightly without deforming the microchannels. The improvement in wettability through UV surface modification technique is advantageous for the bonding behavior between the capping polymer and the polymer containing microfluidic channels, even at lower pressure and lower bonding temperature compared to the polymer bonding without a surface modification process. The raised PMMA surface energy takes more than one month to restore hydrophobicity once the surface energy is modified. However, the IPA rinsing process reported in this study can possibly increase the water contact angle up to 58° immediately, regardless of UV exposure time by simply allowing the IPA solution and DI water to flow.

## Figures and Tables

**Table 1:** Water contact angle of PMMA for non-rinsing samples after UV exposure.

Dose [J/cm^2^]	Water Contact Angle [°]						
	Pristine	0 h	1 h	24 h	7 Days	15 Days	30 Days
1.5	70.9 ± 1.9	55.6 ± 3.1	57.2 ± 3.4	61.3 ± 3.3	63.1 ± 1.4	63.5 ± 1.8	64.2 ± 3.5
3	71.5 ± 2.3	49.9 ± 3.8	51.3 ± 2.7	56.9 ± 2.1	58.8 ± 1.5	59.0 ± 2.3	60.9 ± 2.8
6	71.0 ± 1.9	45.8 ± 1.9	47.4 ± 1.5	52.5 ± 2.4	55.1 ± 2.8	56.0 ± 1.8	58.2 ± 1.9
9	71.2 ± 1.7	43.0 ± 2.5	46.3 ± 6.6	48.2 ± 4.9	50.0 ± 6.0	52.6 ± 4.2	54.2 ± 3.5

**Table 2:** Water contact angle of PMMA for rinsing samples after UV exposure.

Dose [J/cm^2^]	Water Contact Angle [°]							
	Pristine	0 h	Rinsing	1 h	24 h	7 Days	15 Days	30 Days
1.5	70.1 ± 2.6	57.4 ± 3.2	59.3 ± 3.2	64.0 ± 3.2	65.1 ± 3.5	65.8 ± 2.1	66.5 ± 2.1	69.5 ± 2.2
3	69.6 ± 2.5	51.7 ± 2.6	57.6 ± 2.3	61.1 ± 3.4	63.5 ± 2.1	63.6 ± 1.5	64.3 ± 2.0	68.3 ± 2.2
6	70.2 ± 2.1	46.4 ± 2.0	58.1 ± 3.3	61.1 ± 2.8	63.8 ± 3.2	63.7 ± 1.7	64.2 ± 1.8	67.1 ± 2.5
9	70.8 ± 2.3	43.2 ± 1.3	58.2 ± 2.4	59.3 ± 2.4	61.7 ± 3.3	62.36 ± 2.0	62.8 ± 2.3	68.9 ± 2.1

## Data Availability

Not applicable.
